# Access to and perceived value of post-ICU follow-up: an international cross-sectional survey of ICU professionals and adult critical illness survivors

**DOI:** 10.1016/j.aicoj.2026.100141

**Published:** 2026-08-26

**Authors:** Anne-Françoise Rousseau, Susannah Leaver, Kristina Fuest, Victoria Metaxa, Guy François, Bernard Lambermont, Stefan J. Schaller, Richard Rezar, Margo M.C. van Mol, Michael Beil

**Affiliations:** a Department of Intensive Care, University Hospital of Liège, Liège, Belgium; b Research Unit for a Life-Course Perspective on Health & Education-RUCHE, University of Liège, Liège, Belgium; c Department of Intensive Care, St George's NHS Foundation Trust, London, United Kingdom; d Department of Anesthesiology and Intensive Care, TUM School of Medicine and Health, Munich, Germany; e Department of Intensive Care, King’s College Hospital, NHS Foundation Trust, London, United Kingdom; f European Society of Intensive Care Medicine, Brussels, Belgium; g Medical University of Vienna, Department of Anaesthesia, Intensive Care Medicine and Pain Medicine, Clinical Division of General Anaesthesia and Intensive Care Medicine, Vienna, Austria; h Charité - Universitätsmedizin Berlin, Corporate Member of Freie Universität Berlin and Humboldt Universität zu Berlin, Department of Anaesthesiology and Intensive Care Medicine (CCM/CVK), Berlin, Germany; i Department of Cardiology and Intensive Care, Clinic of Internal Medicine II, Paracelsus Medical University Salzburg, Salzburg, Austria; j Department of Intensive Care, Erasmus MC, University Medical Centre, Rotterdam, the Netherlands; k Department of Medicine, NHS Highland, Inverness, United Kingdom

**Keywords:** Post-intensive care syndrome, Follow-up, Survivorship, Patient-centred care, Health service research

## Abstract

**Background:**

Post-intensive care syndrome (PICS) is a frequent and debilitating consequence of critical illness, yet access to structured post-ICU follow-up remains inconsistent.

**Methods:**

We conducted an ESICM-endorsed, international, cross-sectional online survey using two complementary questionnaires to explore the perspectives of ICU healthcare professionals (ICU-HP) and adult ICU survivors (ICU-S), disseminated through ESICM communication channels, professional networks, patient associations, and social media. Both surveys explored post-ICU follow-up practices, access to care, perceived benefits, and unmet needs.

**Results:**

A total of 276 ICU-HP from 242 hospitals across 43 countries and 106 ICU-S from 47 ICUs across 6 countries participated in the survey. Post-ICU follow-up was reported to be available by 45% of ICUs and accessed by 58.5% of patients. Among ICU survivors, 55.7% reported having received information about PICS, while 86.2% of those not informed indicated it would have been useful. Follow-up practices showed marked heterogeneity in patient selection, timing, content, and duration. Among patients who had access to post-ICU follow-up, screening of all three core PICS domains (physical, cognitive and psychological functions) was performed in only 40.7% of cases. Nevertheless, 56.4% reported feeling better following follow-up, mainly due to perceived support and improved understanding of their condition. Among ICU-HPs offering follow-up, 55.1% reported that this activity had led to changes in clinical practice within their ICU, and 97.4% expressed intent to continue their programs although limited training opportunities and a lack of formal guidance were reported by a substantial proportion of respondents.

**Conclusions:**

This survey demonstrates inconsistent access to post-ICU follow-up and information about PICS, despite the perceived value of post-ICU follow-up among both ICU-S and ICU-HP. Improving post-ICU care will require dedicated training and structured guidelines, highlighting the key role of scientific societies in ICU survivorship care.

## Highlights

• Access to information about post-intensive care syndrome (PICS) and to post-ICU follow-up remains inconsistent, with marked international heterogeneity, despite strong interest among ICU survivors and the perceived usefulness of such information and services.
• Patients primarily value relational and informational aspects of post-ICU follow-up, such as being listened to and gaining a better understanding of their ICU experience, rather than symptom resolution alone. This highlights a gap between patient- and clinician-centred outcomes.
• There is a clear need for structured guidance, training, and standardisation, with scientific societies playing a pivotal role in supporting education, guideline development, and coordinated ICU survivorship care.

## Introduction

Survival after critical illness has improved substantially over the past few decades [1]. However, many intensive care unit (ICU) survivors experience new or persistent impairments after hospital discharge [2]. These impairments may be related, at least in part, to persistent inflammation, immune dysfunction, catabolism, and ICU-acquired weakness, which can contribute to prolonged recovery and long-term disability [3]. The long-term physical, cognitive, and psychological sequelae, collectively referred to as post-intensive care syndrome (PICS), may persist for months or years and are associated with reduced health-related quality of life, and major social and familial consequences [4]. The severity, combination, timing of onset, and trajectory of these impairments vary substantially among ICU survivors according to premorbid conditions, ICU exposures, age, social environment, and healthcare systems factors. As a consequence, long-term recovery and quality of life have become key outcomes of critical illness, complementing traditional measures such as short-term survival and mortality [5]. Given the marked heterogeneity in the presentation and trajectory of PICS, its detection and management require a proactive, structured, and longitudinal approach rather than reliance on unstructured, symptom-driven care [6,7].

Early identification of post-ICU sequelae, timely referral to appropriate services, and provision of clear information to patients and relatives are considered essential to optimise recovery trajectories and reduce unmet needs after critical illness [8]. In response to these challenges, post-ICU follow-up pathways and dedicated post-ICU clinics have been developed in an increasing number of healthcare systems [9]. Follow-up programs generally represent one component of a broader survivorship pathway and may include multidisciplinary assessment screening for PICS-related impairments, patient education, reassurance, referral to specialised services, and coordination of subsequent interventions, including rehabilitation when indicated [3]. Beyond patient-centred benefits, post-ICU follow-up has also been proposed as a tool to improve ICU practice through feedback on long-term outcomes, as well as clinician’s well-being [10,11]. Despite increasing interest, post-ICU follow-up remains characterised by remarkable heterogeneity [12], reflecting the limited number of dedicated guidelines and the absence of a universally accepted standard of care. Moreover, evidence supporting the effectiveness of post-ICU follow-up remains inconclusive [13,14]. Consequently, the real or perceived value of post-ICU follow-up remains a matter of debate. This lack of consistent evidence may partly reflect the considerable heterogeneity of post-ICU follow-up models and interventions evaluated to date, rather than the absence of benefit of post-ICU follow-up itself. Differences in patient selection, timing, staffing, assessed domains, interventions delivered, and the structural characteristics of healthcare systems may substantially influence both implementation and outcomes. The absence of standardised processes may limit the comparability of studies and the feasibility of multicentre implementation, while insufficient individualised follow-up pathways may reduce the ability of interventions to address the diverse and evolving needs of ICU survivors.

Studies or surveys exploring the organisation of post-ICU follow-up clinics, screening strategies for PICS, rehabilitation programs, and survivor experiences and needs have predominantly examined healthcare professional perspectives and patient experiences in isolation and have most frequently been conducted within single countries or healthcare systems [[14], [15], [16], [17]]. Consequently, a broader and more integrated understanding of post-ICU follow-up across different healthcare settings, encompassing its availability, organisation, perceived usefulness, and future priorities from both healthcare professional and ICU survivor’s perspectives, is warranted. Incorporating the viewpoints of both healthcare providers and ICU survivors is essential to inform future models of care that are clinically relevant, acceptable, and patient-centred. The CORFU survey was therefore designed to describe the availability, organisation, and perceived benefits of post-ICU follow-up, from the perspectives of both healthcare providers and patients, and to provide pragmatic insights into current post-ICU delivery of care and priorities for future development.

## Methods

### Study design

We conducted an international, cross-sectional survey comprising two complementary online questionnaires: one targeting ICU healthcare professionals (ICU-HP) and the other targeting adult ICU survivors (ICU-S). The study protocol, questionnaires, and dissemination plan received formal endorsement from the European Society of Intensive Care Medicine (ESICM). The study was also approved by the Ethics Committee of the University Hospital of Liege, Belgium (local reference 2025/231, 25th March 2025). We obtained consent from participants at the beginning of the survey.

### Survey reporting

The methodology and findings of the survey are presented in accordance with the Checklist for Reporting Results of Internet E-Surveys (CHERRIES) statement [18].

### Survey development

Both questionnaires were developed iteratively, using a similar method, following commonly accepted principles for healthcare survey design [19]. They were initially drafted by a group of representatives from the ESICM Health Services Research and Outcomes (HSRO) section (AFR, SL, MB), with multiple rounds of refinement. To ensure validity, the pre-final set of questions was further reviewed by a steering committee of other ESICM representatives (KF, for the HSRO section and VM for the Ethics section) for the ICU-HP questionnaire and by representatives from the *ICUsteps* support charity (CJ and 4 former ICU patients) for the ICU-S questionnaire. The questionnaires were adjusted following their feedback. Lastly, the two questionnaires were pretested at the institutions of the two main authors (AFR, SL), by five former ICU patients and by ICU clinicians. Specifically, the pretest used the ESICM Clinical Sensibility Testing Tool, which evaluates several domains on Likert scales, including comprehensibility (ease of understanding and interpretation), face validity (whether the questionnaire appears to measure the intended concepts), relevance (whether the questions address the research objectives without unnecessary redundancy), format and respondent burden (simplicity of response options and acceptability of questionnaire length), and utility (the ability of the survey to generate meaningful information for clinical practice and future research). Comments were provided and a few minor changes were implemented.

The ICU-HP survey assessed demographics, ICU characteristics (including the name of the hospital), availability and organisation of post-ICU follow-up programs, patient selection, timing of follow-up, resources, perceived benefits and barriers, and expectations for future development. It comprised a total of 51 potential questions (see Appendix 1). The questionnaire was made available in English, Spanish, and Portuguese. Additional translations were provided upon request to facilitate dissemination in regions where English is not widely spoken and to optimise response rates. The ICU-S survey explored demographics, ICU stay characteristics, information received about PICS, perceived usefulness of the information, access to follow-up, satisfaction with follow-up content, and views on unmet needs and possible improvements, and comprised 30 potential questions (see Appendix 1). The ICU-S questionnaire was made available in English, French, and Dutch, with the same objective of improving accessibility, as not all eligible ICU survivors spoke English. Most questions were closed-ended, using multiple-choice formats allowing either single or multiple possible responses. A limited number of open-ended questions were included to capture more subjective views and free-text opinions. Not all questions were displayed to all respondents, as adaptive logic was used based on responses to previous items. Consequently, the estimated completion time ranged from approximately 5−30 min.

Translation of the final English versions of the developed questionnaires was performed by healthcare professionals with expertise in intensive care and post-ICU follow-up, who were native speakers of the target language and working in the corresponding countries or regions, in order to preserve the conceptual meaning of questionnaire items while allowing adaptation to local linguistic and cultural contexts.

At the beginning of both questionnaires, definitions of ICU, post-intensive care syndrome (PICS), PICS-family (PICS-F), and post-ICU follow-up were provided to ensure that all respondents shared a common conceptual framework. In the ICU-HP questionnaire, post-ICU follow-up was defined as: “a program ensuring the follow-up of ICU survivors in a structured follow-up clinic, including consultations in an outpatient or one-day setting, scheduled after hospital discharge, run by different types of healthcare providers, and aiming to monitor the evolution after an ICU stay. Models of post-ICU follow-up may vary across Europe.” Respondents were also informed that post-ICU follow-up, in the context of this survey, did not refer to in-hospital weaning units. For the ICU-S questionnaire, a simplified definition of post-ICU follow-up was used: “consultations with healthcare providers especially aimed to monitor the evolution after an ICU stay.”

### Survey dissemination and participants

The ICU-HP survey was open to healthcare professionals working in all types of ICUs, without restriction to adult or paediatric settings, as the questionnaire primarily explored broad organisational and conceptual aspects of post-ICU survivorship care, including program organisation, training, objectives, barriers, and perceived benefits, which may be shared across adult and paediatric settings despite differences between adult and paediatric populations. Conversely, because recruiting adolescent survivors or parents of paediatric ICU survivors across multiple countries is particularly challenging, the ICU-S survey was intentionally restricted to adult survivors.

The ICU-S survey was open to adult ICU survivors whose ICU stay had occurred within the previous two years. This time restriction was applied to limit recall bias regarding patients’ recollection of information received (i.e. any information provided regarding the potential consequences of critical illness and ICU stay, irrespective of the mode of delivery), follow-up experiences, and perceived needs after critical illness, while reducing heterogeneity related to temporal changes in post-ICU follow-up practices and healthcare organisation.

The questionnaires were created using SurveyMonkey® (SurveyMonkey Inc, San Mateo, CA, USA). Participants were pseudo-anonymised using a self-generated identification code composed of the first two letters of their mother’s first name and the first two letters of their father’s surname. Most of the questions were mandatory, aiming to ensure completion. Post-submission review or modification of responses was not allowed. Information about the survey, its purpose and informed consent was explained at the beginning of the online survey. It was a voluntary survey, and there was no incentive to participate.

The surveys were open for participation from May 5th until December 1st, 2025. The survey links were disseminated via multiple channels. The ICU-HP survey was advertised on the ESICM website. In addition, the survey was publicised twice on the different ESICM’s social networks. It was not mandatory to be an ESICM member to take part in the survey. We opted for individual responses rather than a single ICU-wide response to account for potential differences in perception among healthcare providers within the same unit. The ICU-S survey was distributed through national and hospitals patients’ associations. For both surveys, the links were shared by the investigators and collaborating colleagues via professional mailing lists, institutional emails, and social media networks. This dissemination strategy relied partly on snowball sampling, whereby recipients were encouraged to forward the survey links to other eligible ICU healthcare professionals or ICU survivors.

### Data analysis

Data from the questionnaire were exported from SurveyMonkey® and stored as an Excel file (Microsoft Corp, Redmond, WA). Duplicates from the same participant were identified and excluded. Questionnaires were considered valid when informed consent was provided, demographic data were completed, and respondents answered the question regarding the availability/access to post-ICU follow-up, corresponding to a key objective of the survey. For ICU-HP, analyses related to follow-up program additionally required respondents to report being involved in or aware of the post-ICU follow-up program. For ICU-S, analyses related to a follow-up program required respondents to report having had access to a structured post-ICU follow-up organised by their ICU. For the purpose of this survey, ICU survivors were considered to have had access to post-ICU follow-up if they reported having attended at least one structured post-ICU follow-up consultation organised by their ICU after hospital discharge. Partially completed questionnaires fulfilling these criteria were retained for analysis. Although most questions were mandatory, respondents remained free to stop the survey at any stage, resulting in incomplete questionnaires. Missing data were not replaced, resulting in variable denominators across analyses depending on the number of respondents answering each specific question.

Given the exploratory nature of the survey, analyses were primarily descriptive, using Graphpad Prism (version 10.6.1 for Mac OS, Graphpad Inc., San Diego, CA, USA). Categorical variables are presented as counts and percentages. Percentages were calculated using the number of respondents who answered the corresponding question as the denominator. Continuous variables are presented as median and interquartile range. Free-text responses were reviewed independently by the study investigators to identify recurrent themes and commonly reported suggestions. Given the exploratory nature of the survey and the limited amount of qualitative data collected, no formal qualitative analysis or thematic coding framework was applied.

## Results

### Characteristics of respondents and intensive care settings

**Fig. 1:**
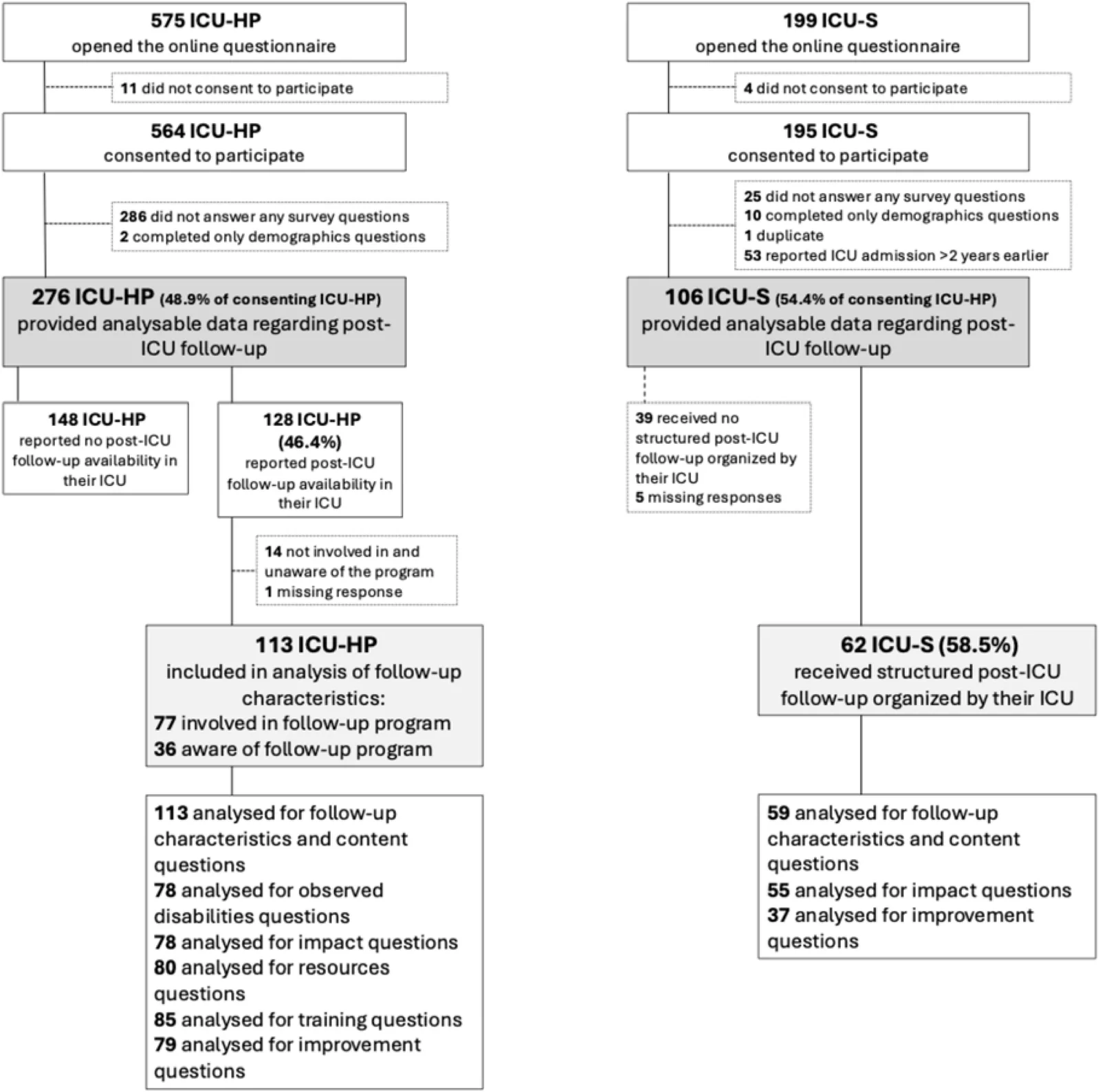
Flow chart of participant inclusion and analytical samples for the ICU healthcare professional (ICU-HP) and ICU survivor (ICU-S) surveys.

Out of the 575 respondents who opened the ICU-HP online survey, 11 declined to participate, 286 consented but did not answer any survey questions, and 2 completed only demographic questions. A total of 276 ICU-HP from 242 different hospitals and 43 different countries provided analysable data regarding post-ICU follow-up (see flow chart, Fig. 1).

Out of 199 patients who opened the ICU-HP online survey, 4 declined to participate, 25 consented but did not complete any survey questions and 53 reported an ICU stay that had occurred more than two years prior and were therefore excluded. Ten respondents completed only demographic questions, and one duplicate response was excluded. A total of 106 patients reported an ICU admission to 47 different ICUs during the last 2 years and provided analysable data regarding post-ICU follow-up (see flow chart, Fig. 1). These 106 ICU survivor responses originated from 6 countries, with the majority of respondents coming from Belgium, France, Germany, and the United Kingdom.

**Fig. 2:**
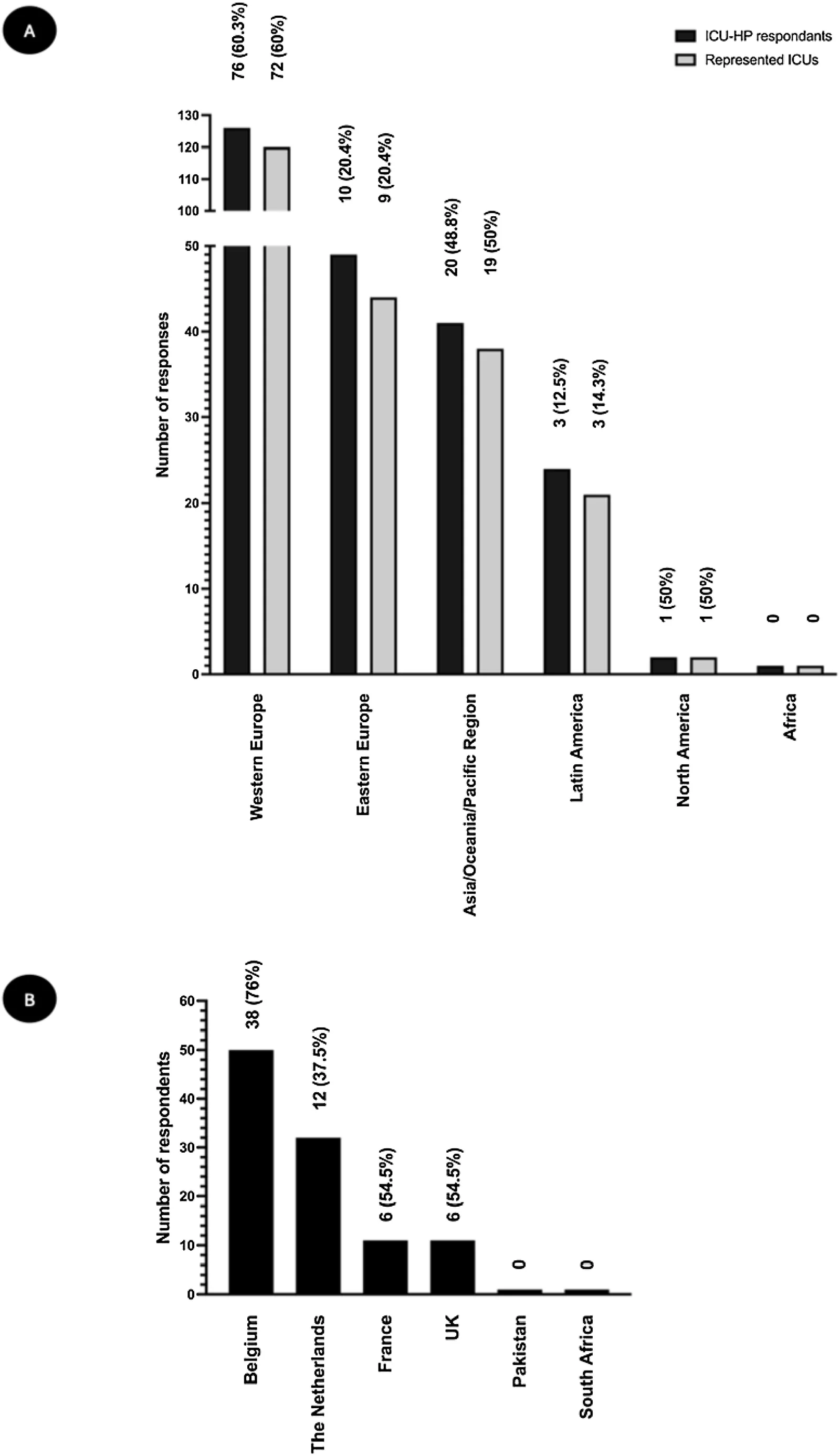
Geographical distribution of survey respondents and post-ICU follow-up programs. The Fig. 2A shows the geographical distribution of ICU-HPs, which is presented according to individual respondents (black bars) and represented ICUs (grey bars). Respondents were from 43 different countries. Geographical regions were grouped for descriptive and figure readability purposes only and should not be interpreted as reflecting homogeneous healthcare systems or post-ICU follow-up practices. The Fig. 2B shows the geographical distribution of ICU-S respondents, from 6 different countries. In each Figure, counts and proportions displayed at the top of the bars represent post-ICU follow-up programs reported as available according to individual ICU-HP responses (black bars) or represented hospitals (grey bars) in Fig. 2A, or according to ICU-S in Fig. 2B, within each country or geographical region. HP: healthcare providers; ICU: intensive care unit; S: survivors.

Fig. 2 and Supplemental Table S1 depict the geographical distribution of survey participants across countries. The characteristics of the respondents are detailed in Table 1. Briefly, approximately half of ICU-HP respondents were physicians, most worked in university or university-affiliated hospitals, and the majority practiced in mixed medical-surgical ICUs. ICU-S respondents generally reported prolonged ICU stays and were most frequently admitted to university or university-affiliated hospitals.

**Table 1:** Characteristics of the participants.

Variable		Total
ICU-HP survey (n = 276)		
Profession	ICU physician (specialist)	141 (51.2)
	ICU physician (in training)	15 (5.4)
	ICU physician (head)	51 (18.6)
	ICU nurse	32 (11.6)
	ICU advanced clinical practitioner	11 (3.9)
	ICU head nurse	5 (1.8)
	ICU physiotherapist	11 (3.9)
	other	10 (3.6)
Type of hospital	University hospital	125 (45.3)
	Teaching hospital of a university	65 (23.5)
	Non-university public hospital	62 (22.5)
	Private hospital	24 (8.7)
Patients admitted in the ICU	Adults	234 (84.9)
	Children	11 (3.9)
	Both adults and children	31 (11.2)
Type of ICU	Mixed ICU	212 (76.9)
	General medical ICU	45 (16.3)
	Specialised medical ICU	2 (0.7)
	General surgical ICU	11 (3.9)
	Specialised surgical ICU	6 (2.2)
Number of ICU beds		16 (10−24)
ICU-HP-reported average ICU length of stay (days)		5 (4−8)
ICU-S survey (n = 106)		
Age (years)		55 (43−63)
Type of ICU	University / teaching hospital	69 (65.1)
	Non university / teaching hospital	30 (28.3)
	Don’t know	7 (6.6)
Self-reported duration of the ICU stay (days)		15 (10−29)

Data are expressed as numbers and percentages or median (P25-P75).

HP: healthcare providers; ICU: intensive care unit; S: survivors.

### Provision of information on post-intensive care syndrome

Among the 106 ICU-S who completed the questionnaire, 18 (16.9%) patients reported that they did not remember whether they had received information regarding PICS, while 59 (55.7%) reported having received such information. Most of them (53/59, 89.8%) indicated that this information was beneficial. The source was predominantly from the ICU or the hospital (50/59, 84.7%). In five cases, patients reported obtaining the information through social media platforms or patient associations rather than directly from healthcare professionals.

Conversely, among the 29 patients who did not receive information about PICS, the majority (25/29, 86.2%) reported that such information would have been beneficial. They reported that the content of such information should help them better understand the post-ICU clinical condition they experienced and provide practical guidance, including useful contact details for healthcare institutions and other resources. Among the free-text comments, two illustrative comments selected for their relevance to the topic of information delivery suggested that information about PICS should be provided in the presence of relatives. The preferred format of information delivery would have been an oral discussion (31/59, 52.5%), potentially supplemented by written material (14/59, 23.7%), and preferably provided during the ICU stay (47/59, 79.7%) rather than after ICU discharge (12/59, 20.3%).

### Availability of post-ICU follow-up programs and patient access to follow-up

Among the 242 different hospitals represented in the survey, 109 (45%) ICUs reported offering a post-ICU follow-up program. These services were available in 30 of the 43 countries represented (69.8%). Their geographical distribution across responding countries is shown in Fig. 2 and in the Supplemental Table S1. Reported availability of post-ICU follow-up appeared heterogeneous across responding geographical regions, with respondents from Western Europe more frequently reporting the availability of dedicated post-ICU follow-up programs than respondents from Eastern Europe, while more similar proportions were observed in the Asia/Oceania/Pacific region and Latin America. Post-ICU follow-up programs were reported by 77/159 (48.4%) university or university-affiliated teaching hospitals, and by 32/81 (39.5%) non-university public or private hospitals.

From the patient perspective, 62/106 (58.5%) ICU-S reported having had access to a structured post-ICU follow-up program organised by their ICU. Among ICU survivors who did not benefit from post-ICU follow-up (39/106, 36.8%), most of them (27/39, 69.2%) perceived that such follow-up would have been useful. The perceived usefulness of post-ICU follow-up in this subgroup is presented in Supplemental Fig. S1. A minority (10/39, 25.6%) reported no perceived need for structured follow-up. Notably, among this minority, 60% had not received prior information about PICS.

### Characteristics and content of post-ICU follow-up visits

Among ICU-HP reporting the availability of post-ICU follow-up in their ICU, 113 were involved in or aware of the program (see flow chart, Fig. 1). This subgroup served as the analytical sample for questions on follow-up characteristics. Key characteristics of post-ICU follow-up programs (patient selection criteria, timing of program initiation, timing of the first follow-up visit, visit modalities, number of visits, and timing of the final follow-up visit) are detailed in Supplementary Fig. S2. Briefly, patient selection was most commonly based on ICU length of stay (53/113, 46.9%) or the need for organ support during the ICU stay (42/113, 37.2%), whereas unrestricted access for all ICU survivors was less frequently reported (12/113, 10.6%). Follow-up programs were most often initiated at ICU discharge (56/113, 49.6%). The first follow-up visit was most commonly scheduled at 3 months after ICU discharge (37/113, 32.7%). In-person consultations represented the predominant modality of follow-up (61/113, 54%). Most programs offered repeated visits according to patient needs (51/113, 45.1%).

Patients who had access to post-ICU follow-up within dedicated structures also reported marked heterogeneity in follow-up organisation, and in the domains that were explored (Supplemental Fig. S3). Screening covering all three core PICS domains simultaneously (physical, psychological, and cognitive) was performed in only 24/59 (40.7%) patients, while a more comprehensive assessment—including the three core domains plus swallowing, nutrition, sleep, dependency, and quality of life—was conducted in only 8/59 (13.6%) patients.

During follow-up, 51/59 (86.4%) patients reported having had the opportunity to discuss their lived experience of the ICU stay, and only 2/59 (3.4%) patients reported deliberately choosing not to do so. Overall, 47/59 (79.7%) patients considered that all their problems had been addressed during follow-up. Among those who did not share this view, respondents expressed a need for better support for relatives and improved information regarding available resources to enhance their care.

The most frequent disability detected during post-ICU follow-up by ICU-HPs is detailed in Supplemental Fig. S4: the most observed impairment was physical disability, reported by 22/78 (28.2%) ICU-HP respondents.

### Perceived impact of post-ICU follow-up

**Fig. 3:**
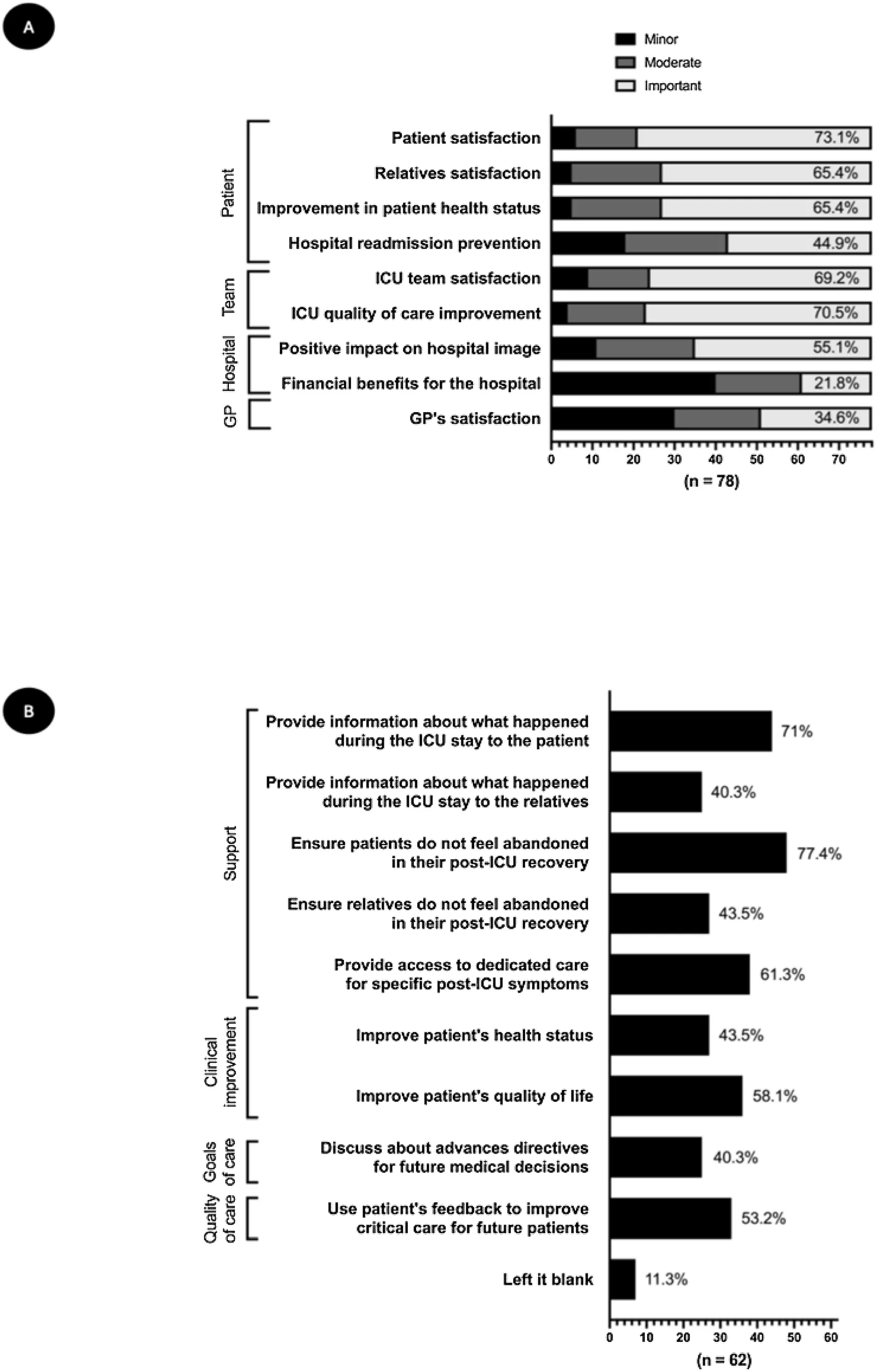
Perceived impact and objectives of post-ICU follow-up. (A) ICU-HPs’ perceptions of the potential benefits of post-ICU follow-up programs, graded from minor to major impact. Numbers displayed within the bars indicate the proportion (%) of ICU-HPs (out of the total sample of 78 respondents) rating each item as having a major impact. (B) Primary objectives of a well-conducted post-ICU follow-up according to ICU-S (closed-ended questions with multiple responses allowed). GP: general practitioner; HP: healthcare providers; ICU: intensive care unit; S: survivors.

A total of 78 ICU-HP completed this section. Their perceptions of the potential benefits of post-ICU follow-up programs are presented in Fig. 3. Overall, ICU healthcare professionals most frequently considered post-ICU follow-up to have an important impact on patient satisfaction, ICU quality of care improvement, and ICU team satisfaction. These perceived benefits were accompanied by reported changes in clinical practice within the ICU in 55.1% (43/78) of cases, most commonly involving sleep management, delirium prevention and management, nutritional care, and approaches to goals of care. Consistent with these perceived benefits, nearly all respondents (76/78, 97.4%) indicated their intention to maintain existing post-ICU follow-up programs. By contrast, benefits related to hospital finances and general practitioners’ satisfaction were less frequently perceived as important outcomes.

Patients’ perceptions of the impact of post-ICU follow-up on their recovery trajectory are presented in Supplemental Fig. S5. The primary objectives for ICU-S of a well-conducted post-ICU follow-up are presented in Fig. 3. Briefly, 31/55 (56.4%) ICU-S reported feeling better afterwards, mainly because they felt listened to and gained a better understanding of their recovery. The three most valued aspects of post-ICU follow-up for them were avoiding feelings of abandonment, understanding the ICU experience, having a facilitated access to dedicated care for post-ICU symptoms.

### Resources and training

**Fig. 4:**
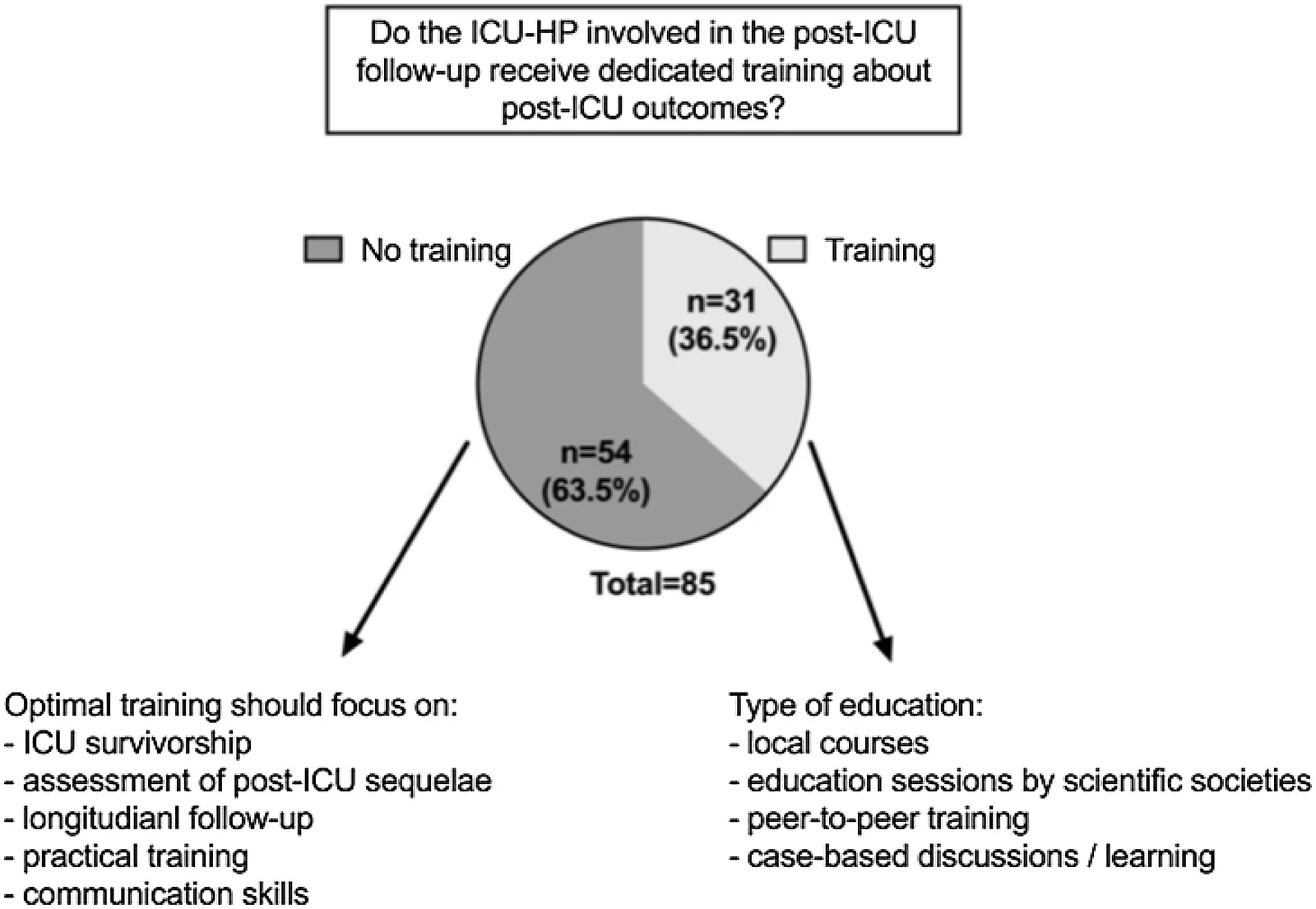
Training of ICU-HPs involved in post-ICU follow-up programs, including existing training formats and suggested training topics (open-ended question). Data are expressed as counts and percentages. HP: healthcare provider; ICU: intensive care unit.

A total of 85 ICU-HP completed the section related to training. Most of the respondents (82/85, 96.5%) reported that at least one ICU team member was directly involved in post-ICU follow-up activities. Training received by healthcare professionals involved in post-ICU follow-up, as well as perceived training needs, are summarised in Fig. 4. In parallel, 58/85 (68.3%) reported insufficient training among other stakeholders involved in the post-ICU care pathway, citing both limited skills and a lack of awareness regarding post-ICU outcomes.

Existing guidelines informed follow-up practices in 40% of respondents (32 of the 80 participants who answered this section). These included national guidelines for post-ICU follow-up, such as Portuguese guidelines or National Institute for Health and Care Excellence (NICE) recommendations, as well as the Life after Critical Illness guidance developed by the Faculty of Intensive Care Medicine in the United Kingdom. Some respondents also reported relying on non-dedicated guidelines, such as the European Society for Clinical Nutrition and Metabolism (ESPEN) or the Guidelines for the Provision of Intensive Care Services (GPICS). The majority of respondents (73/80, 91.2%) indicated that they would modify the format of their post-ICU follow-up if international guidelines were to be published.

Collaboration with primary care services was described by 80 ICU-HP respondents and is detailed in Supplemental Figure S6. The majority of respondents (75/80, 93.8%) reported that general practitioners (GPs) lacked sufficient understanding of post-ICU health issues. This limitation was attributed to GPs’ limited exposure to ICU survivors and their specific needs. Suggested approaches to improve GPs’ knowledge included enhancing the quality and clarity of written communication addressed to them, providing training opportunities within post-ICU clinics, fostering closer collaboration between ICU teams and primary care, and making dedicated post-ICU care guidelines available.

Funding for post-ICU follow-up is detailed in Supplemental Figure S7.

Several respondents indicated that data from post-ICU follow-up were systematically collected, either in local databases (52/80, 65.0%), in regional registries (2/80, 2.5%) or in national registries (2/80, 2.5%).

### Suggested improvements for post-ICU follow-up

**Fig. 5:**
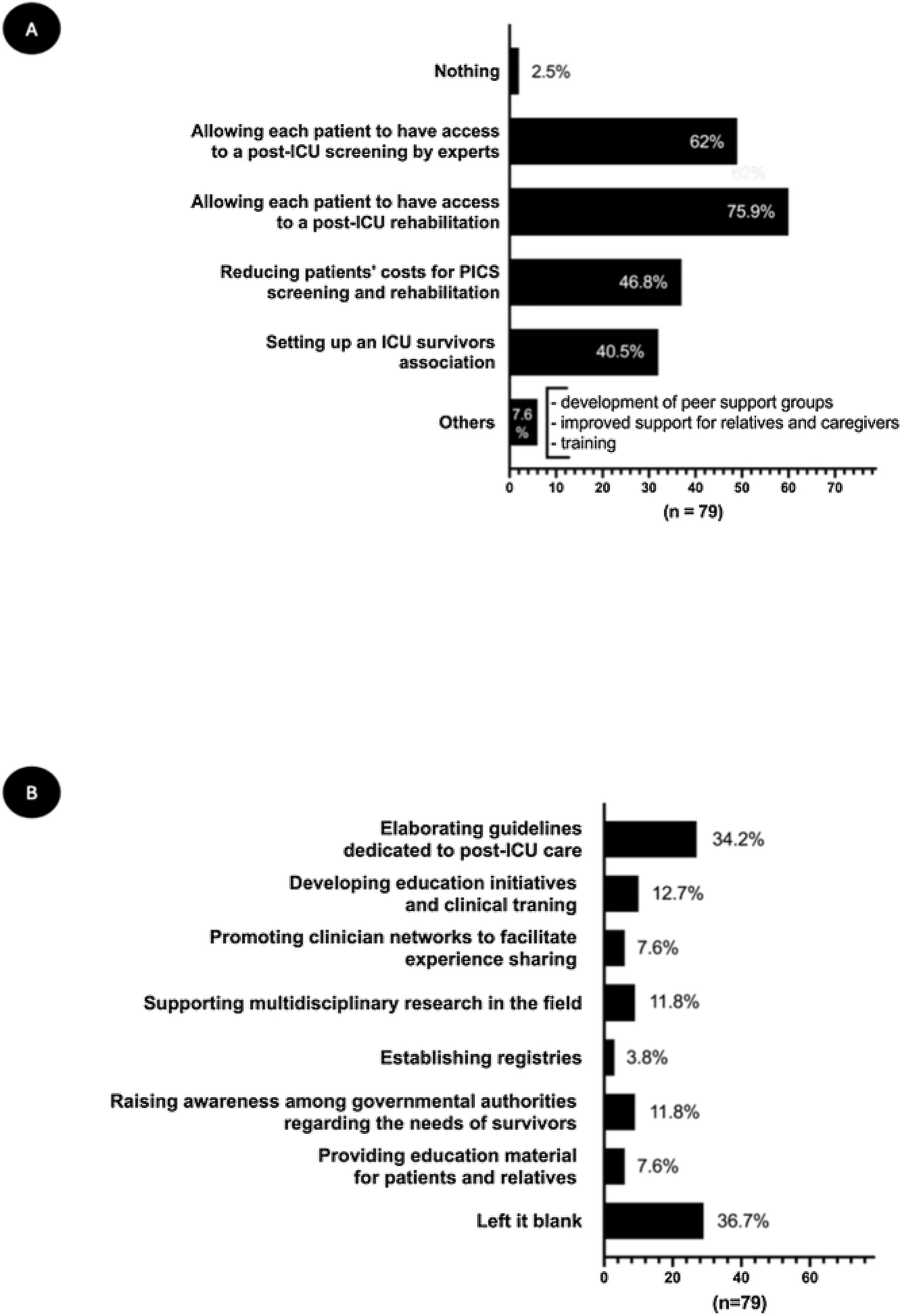
Areas of improvement in the care of ICU survivors (A) and key roles of intensive care scientific societies in that domain (B), identified by the ICU-HP respondents (closed-ended questions with multiple responses allowed). Numbers displayed within or beside the bars indicate the proportion of ICU-HP (out of the total sample of 79 respondents) who selected each answer (A) or who suggested each item (B). ICU: intensive care unit; PICS: post-intensive care syndrome.

A total of 79 ICU-HP identified areas for improvement, which are described in Fig. 5. Fifty ICU-HP respondents identified key roles that scientific societies—and notably the European Society of Intensive Care Medicine (ESICM)—must play in improving post-ICU care (Fig. 5).

ICU-S provided insights into how the medical community could improve care for ICU survivors. Patients emphasised that the recovery after an ICU stay extends beyond physical sequelae, highlighting the psychological and emotional dimensions of recovery. One respondent also considered post-ICU follow-up relevant even if its benefits were not immediately apparent. Their suggestions are described in Supplemental Table S2.

## Discussion

This ESICM-endorsed survey provides a broad overview of post-ICU follow-up practices and perceived benefits, integrating the parallel perspectives of ICU-HP across 43 countries and ICU-S from 6 countries, on a topic of growing importance for patients, clinicians, and healthcare systems. It highlights both similarities and discrepancies in expectations, experiences, and perceptions of the value of post-ICU care.

Several key findings emerge from this survey. First, from the patient perspective, information about PICS and post-ICU trajectories remains insufficient. Nearly half of ICU-S reported not having received information about PICS, despite expressing a strong interest in such information perceiving it as useful. This lack of information, which has been highlighted for years [15], contributes to uncertainty, unmet needs, and feelings of abandonment after ICU discharge—issues repeatedly emphasised by patients [17,20]. Second, less than half of responding ICUs reported offering structured post-ICU follow-up, and a similar proportion of patients reported having access to such care. This suggests that access to post-ICU follow-up remains heterogeneous across responding healthcare systems and institutions, a finding that has been consistently reported in previous surveys involving different regions and stakeholders’ groups [[21], [22], [23]]. Notably, both patients who did not receive follow-up and those who did not receive information about PICS frequently reported that these interventions would have been beneficial for them, underscoring a gap between current provision and patient expectations. Third, when post-ICU follow-up exists, it is characterised by marked heterogeneity in organisation and content. Selection criteria, timing of initiation, number and scheduling of visits, professional involvement, and assessment domains vary widely across centres. This heterogeneity is not a novel finding, as wide variability in the availability, organisation, and content of post-ICU follow-up has been consistently reported across healthcare systems in surveys and reviews [21,24], reflecting differences in resources, local priorities, and the absence of universally accepted standards.

A key finding of this survey is that PICS assessment during post-ICU follow-up remains inconsistent and incomplete across many centres. Fewer than half of patients underwent screening of all three core PICS domains—physical, cognitive, and psychological—and comprehensive assessments incorporating additional relevant domains were rare. This stands in clear contrast to ICU-HP’s reports of frequently encountered post-ICU sequelae across multiple domains, highlighting a persistent gap between recognised clinical needs [25,26] and implemented evaluation practices. While factors explaining why systematic screening across all PICS domains is not routinely performed have not been specifically studied in the present survey, they may include limited human resources, time constraints, lack of dedicated multidisciplinary expertise, lack of understanding or awareness of the importance of PICS-related problems, or concerns regarding the cost and feasibility of extended assessments. A pragmatic way to address this gap could be a two-step approach: an initial brief, standardised screen tool covering the core domains for all patients, followed—if screening is positive—by referral to specialised services for more comprehensive and multidisciplinary evaluation [27]. Such a model could balance feasibility with clinical thoroughness while promoting more equitable and structured post-ICU care pathways.

Despite the incomplete and inconsistent screening of PICS domains, ICU survivors generally reported positive experiences and perceived benefits associated with post-ICU follow-up, a finding that aligns with previous reports [28,29], and may reflect perceived support rather than the completeness of formal assessment. Patients primarily attributed this benefit not to symptom resolution or functional improvement, but to being listened to, receiving clear explanations of their ICU experience, and feeling supported throughout their recovery trajectory. These findings highlight a fundamental difference in perspective between clinicians and patients: while healthcare professionals tend to conceptualise post-ICU follow-up through the lens of dysfunction, screening, and measurable clinical outcomes, patients appear to value relational, informational, and emotional dimensions of care more strongly. It also suggests that traditional clinical metrics may be insufficient to capture the impact of post-ICU care, and that patient-centred outcomes—such as reassurance, understanding, continuity, and perceived support—should be more explicitly considered when designing, evaluating, and benchmarking post-ICU follow-up models. Importantly, these apparently divergent perspectives should not be viewed as opposing, as the ultimate goal of post-ICU follow-up remains shared: to support patients in regaining their pre-ICU level of activity, restoring quality of life, facilitating social and professional reintegration, limiting healthcare utilisation, and preventing long-term functional decline. We are nevertheless aware that, in healthcare systems facing constrained resources, it may not be feasible to fully address all dimensions of post-ICU needs. The key challenge will therefore be to identify an optimal allocation of available resources, prioritising interventions that yield the greatest meaningful impact for patients while remaining sustainable and equitable.

From the ICU-HP’s perspective, post-ICU follow-up was infrequently associated with significant changes in ICU practices during the stay. Although more than half of respondents reported some practice modifications—mainly related to sleep, delirium, nutrition, or goals of care—post-ICU follow-up was not perceived as a strong driver of systematic practice transformation. These findings suggest that, despite increased awareness of long-term ICU outcomes, substantial efforts are still required to translate insights gained through post - ICU follow up into routine ICU practice [30]. Nevertheless, all ICUs offering post-ICU follow-up expressed a clear intention to maintain their programs, suggesting a perceived intrinsic value that extends beyond measurable short-term outcomes.

The perceived benefits reported by ICU survivors, together with ICU-HPs’ strong willingness to maintain their follow-up programs, should nevertheless be considered in the context of evidence from randomised controlled trials, which has not consistently demonstrated improvements in conventional clinical outcomes following structured post-ICU follow-up [13]. These findings do not necessarily contradict the positive experiences reported in our survey: the substantial heterogeneity across follow-up models and interventions, survivor populations, healthcare contexts, and outcomes assessed, currently limits the ability to generate robust and generalizable evidence regarding the effectiveness of post-ICU follow-up.

Our findings reveal substantial gaps in both training and guidance among healthcare professionals involved in post-ICU follow-up. The limited availability of dedicated education (particularly during training [31]) may contribute to insufficient awareness and variable knowledge regarding post-intensive care syndrome and survivorship care, potentially leading to differences in patient identification, assessment, referral pathways, and follow-up delivery. In addition, the lack of structured international guidelines provides little in the way of a common framework to support program development and implementation, likely further contributing to the marked heterogeneity observed across post-ICU follow-up models. Importantly, most respondents indicated that their follow-up practices would be modified if such guidance were available, underscoring the pivotal role of scientific societies in developing guidelines, promoting education, and shaping future post-ICU models of care by supporting multicentre research and registries. Beyond the professional sphere, respondents also emphasised the importance of scientific societies in raising awareness among healthcare authorities to secure sustainable funding for ICU care, as well as in supporting patients and families through education and awareness initiatives.

This survey has several important strengths. It provides a large-scale assessment of post-ICU follow-up that integrates the parallel perspectives of ICU-HP and ICU-S, enabling a unique cross-validation of needs, expectations, and perceived impacts. Importantly, the perceived usefulness of post-ICU follow-up reported by both ICU survivors and healthcare professionals should not be interpreted as evidence of clinical efficacy. The present survey assessed perceptions, experiences, and expectations rather than objective outcomes: the impact of post-ICU follow-up on PICS burden, health-related quality of life, functional recovery, social reintegration, and healthcare utilisation requires dedicated prospective outcome-based evaluation. The broad international participation, encompassing respondents from many diverse countries and healthcare systems, captures real-world heterogeneity in post-ICU care. Finally, the ESICM endorsement and rigorous survey development process strengthen the credibility and relevance of the results. However, the study also has several limitations that should be acknowledged. First, as with any cross-sectional, voluntary survey, the results are subject to selection bias. Participants with a particular interest in ICU survivorship and post-ICU follow-up may have been more likely to respond, potentially leading to an overestimation of the availability of post-ICU follow-up services and of their perceived importance. This bias may have been particularly pronounced among ICU survivors, as dissemination through patient associations and dedicated survivor networks likely preferentially reached individuals personally concerned by post-ICU sequelae and recovery issues. Conversely, awareness and prioritisation of post-ICU survivorship remain heterogeneous among healthcare professionals, and post-ICU care is not yet universally perceived as a clinical priority across healthcare systems and ICU teams, as previously reported [21]. This may partly explain the higher proportion of incomplete questionnaires among ICU healthcare professionals compared with ICU survivors. In addition, online surveys are generally associated with lower response rates, which represents an intrinsic methodological limitation [32]. This may further limit the generalisability of the findings to the broader populations of ICU-HP and ICU-S. Furthermore, the exclusively online format may have preferentially reached individuals with greater digital access and literacy. Despite the availability of the questionnaires in several languages, language-related selection bias cannot be excluded, as individuals unable to complete the survey in one of the available languages may have been underrepresented. Beyond these intrinsic limitations, additional structural barriers must be acknowledged, including the difficulty of identifying and reaching ICU survivors after discharge, particularly in settings where structured post-ICU pathways and dedicated patient associations are lacking. Second, the unequal geographical distribution of respondents, particularly among ICU survivors, limits the interpretation and generalisability of regional comparisons. Consequently, geographical differences in post-ICU follow-up availability and practices should be interpreted cautiously and considered exploratory rather than representative of entire healthcare systems or regions. In addition, low-resource settings were likely underrepresented among respondents. Third, the sampling strategy relied partly on dissemination through professional networks, patient associations, and social media, using a snowball approach. As a result, the survey does not allow calculation of a response rate, and the representativeness of respondents across countries, institutions, and healthcare systems cannot be guaranteed. The number of responses varied substantially between countries, limiting the ability to draw robust country-level comparisons. Fourth, the ICU-S survey relied on self-reported data, which may be subject to recall bias, particularly regarding information received during the ICU stay or shortly after discharge. Fifth, the ICU-HP survey captured individual perceptions rather than unit-level practices. Although this approach was intentionally chosen to avoid assuming that a single respondent could represent all healthcare professionals working within a given ICU and to allow the capture of differing perspectives from the same institution, most participating hospitals were ultimately represented by a single respondent. Consequently, within-unit variability could not be meaningfully explored, and discrepancies may exist between reported perceptions and actual institutional policies or practices. Finally, several factors influencing the implementation and organisation of post-ICU follow-up programs, including workforce availability, integration with other healthcare services, and characteristics of the healthcare systems in which respondents were practicing, were not specifically explored. Collecting such information would have provided a more detailed understanding of the observed heterogeneity but would also have substantially increased questionnaire complexity and respondent burden.

## Conclusion

This survey, including respondents from multiple countries, shows that both clinicians and ICU survivors value post-ICU follow-up, although its availability remains inconsistent and its delivery remains highly heterogeneous. Patients report high perceived value, mainly related to support, understanding, and continuity of care, despite incomplete and non-standardised assessment of PICS. The divergence between clinician- and patient-centred perspectives highlights the need to better align post-ICU care with outcomes that matter to patients. Persistent gaps in training and guidance, alongside clinicians’ willingness to adapt practices, emphasise the key role of scientific societies in developing guidelines, education initiatives, and collaborative research to improve ICU survivorship care.

## Author’s contributions

AFR, SL, KF, VM, MVM, MB: conceptualisation and questionnaire design; AFR, MVM translation of the questionnaire (French and Dutch); AFR, GF: methodology; AFR, GF, RR: survey dissemination, AFR: data cleaning, statistical analyses, and interpretation of results; AFR: writing – original draft; SL, KF, VM, BL, SS, RR, MVM, MB: writing – review and editing.

## Consent for publication

Not applicable.

## Ethics approval and consent to participate

This study was approved by the Central Ethics Committee of the University Hospital of Liège, Belgium on March 25th, 2025, number # 2025/231. Consent from participants was obtained at the beginning of the survey.

## Declaration of Generative AI and AI-assisted technologies in the writing process

ChatGPT 5.1 was used to revise the original draft written by AFR for language and style. The language generated by the GAI tool was verified by the co-authors.

## Funding

The present study has not been supported by any funding.

## Availability of data and material

The datasets used and/or analysed during the current study are available from the corresponding author on reasonable request.

## Declaration of competing interest

All authors declare that they have no competing interests.
