# Supplementary material for: Access to and perceived value of post-ICU follow-up: an international cross-sectional survey of ICU professionals and adult critical illness survivors
Source: Ann Intensive Care. 2026 Aug 26;16:100141. doi: 10.1016/j.aicoj.2026.100141 (PMC13564131; doi:10.1016/j.aicoj.2026.100141)
Supplement: Supplementary file 2 [file mmc1.docx]

**Access to and perceived value of post-ICU follow-up:**

**an international cross-sectional survey of ICU professionals and**

**adult critical illness survivors.**

Appendix: CORFU ICU-HP and ICU-S questionnaires

1/ ICU-HP questionnaire

Page 1

CORFU survey

Access to and perceived benefits of post-ICU follow-up in Europe: the perspective of ICU healthcare providers

**Dear Colleagues,**

**We kindly invite you to participate in a survey titled “CORFU survey”.**

**This survey, endorsed by the ESICM, is run by the HSRO section.**

**This ESICM HSRO section is dedicated to outcome research.**

**This survey aims to depict the current practices in terms of post-ICU follow-up**

**and post-intensive care syndrome screening across Europe and to assess the perceived benefits of PICS-targeted post-ICU follow-up.**

**The answers to these questionnaires will be helpful to prepare further dedicated recommendations or expert consensus on the topic.**

**Depending on your current practices, the survey takes about 5 to 25 minutes to complete.**

We are seeking participation from ICU healthcare providers in Europe. ***If you are not a European ICU healthcare provider, please do not complete the survey.***

**Participating in this survey does not expose you to any risk: participation is voluntary and anonymous.**

This research has been reviewed by an Ethical Committee of the University Hospital of Liège (Belgium). Refusal or choice not to participate will not result in any penalty.

The principal investigator, the research team and the sponsor undertake to comply with the regulations on the protection of natural persons in relation to the processing of personal data. The information collected in this survey will be stored securely as long as there is a need for this research purpose. The collected data will not be used or distributed for other future projects.

If you have any questions about your rights as a research participant at any time before, during or after participation, please contact the study team (principal investigator, Pr Anne-Françoise ROUSSEAU, afrousseau@chuliege.be).

**We thank you for your collaboration,**

**On behalf of the HSRO section,**

**Anne-Françoise Rousseau, Michael Beil and Susannah Leaver**

Would you like to proceed with this anonymous survey?

YES / NO

If NO: go to page 10

Page 2

Please read the following abbreviations and definitions before proceeding with the survey:

ICU: intensive care unit

specialised ward in the hospital, where a multidisciplinary team, made up of highly skilled intensive care doctors, nurses, and other healthcare providers, ensure 24-hour critical care for patients at risk of or presenting with one or more organ failure.

PICS: post-intensive care syndrome

new onset or worsening of impairment(s) in physical, cognitive, and/or mental health that arise after the ICU and persists beyond hospital discharge, affecting daily function and quality of life

PICS-F: post-intensive care syndrome family

PICS affecting relatives of a critically ill patient

Post-ICU follow-up:

program ensuring the follow-up of ICU survivors in a structured follow-up clinic, including consultations in an outpatient or a one-day setting, scheduled after hospital discharge, run by different types of healthcare providers, and aiming to monitor the evolution after an ICU stay. Model of post-ICU follow-up may vary across Europe.

PLEASE NOTE that the post-ICU follow-up, in this survey, does not refer to in-hospital weaning units.

Don't worry about the question numbers; your responses will determine the order.

* mandatory questions

Page 3

* 1/ Your initials (the first two letters of your mother’s first name and the first two letters of your father’s surname)

* 2/ What is your profession? Please select a single answer

ICU physician (in training)

ICU physician (specialist)

ICU head of department

ICU nurse

ICU advanced clinical practitioner

ICU head nurse

ICU physiotherapist

Other (please specify below)

+ free text : please specify here

* 3/ Where is your ICU located (city / town)?

* 4/ What country is your ICU located in?

* 5/ What is the name of your hospital?

* 6/ What type of hospital is your ICU located in? Please select a single answer.

University hospital

Teaching hospital associated with a university

Non university public hospital

Private hospital

* 7/ Who is admitted to your ICU? Please select a single answer.

Adults

Children

Both adults and children

* 8/ What type of ICU do you work in? Please select a single answer.

Mixed medical and surgical

Medical – all type of disease

Medical – specific disease only (please specify below)

Surgical – all type of surgeries

Surgical – specific surgery only (please specify below)

+ free text : please specify here

9/ How many beds equipped for mechanical ventilation are there in your ICU?

10/ How many patients were admitted to your ICU in 2024?

11/ What is the median ICU length of stay in your ICU?

Page 4

*12/ Does your ICU offer a post-ICU follow-up clinic in any format?

YES / NO

>> if YES: go to page 5 and question 15

>> if NO: go to question 13

*13/ In your opinion, why does your ICU not offer such follow-up? Please select one or more reason listed below:

I don’t know

It is not currently viewed as a priority in my ICU

There is not enough evidence to support this development

There is an absence of guidelines about post-ICU follow-up

We have not had enough training to provide follow-up

There are insufficient resources available (please specify below)

Follow-up is done by other specialists

Follow-up is done by general practitioners

Other (please specify below)

+ free text : please specify here

*14/ Does your ICU plan to offer a post-ICU follow-up within the next 12 months?

YES / NO

END of the survey: go to page 10

Page 5

*15/ Are you personally involved in this follow-up?

YES / NO

>> if YES: go to page 6 and question 17

>> if NO: go to question 16

*16/ Are you aware of the structure and organisation of this follow-up?

YES / NO

>> if NO: go to page 9 and question 47

>> if YES: go to page 6 and question 17

Page 6

*17/ What criteria are used to select patients for follow-up in your ICU? Please select one or more answer below

No selection, all patients discharged alive from ICU are invited

No selection, all patients are welcome upon demand

Pre-admission health status (please specify below)

Initial critical illness (please specify below)

Selection based on ICU complications (please specify below)

Selection based on the ICU length of stay (please specify the cut-off below)

Selection based on the type of organ support that the patient received (please specify below)

Selection based on post-ICU status (please specify below)

Selection based on post-ICU treatment plan (please specify below)

Selection based on patient’s language

Selection based on patient’s physical distance from the hospital

Only patients included in a clinical trial

Other (please specify below)

+ free text : please specify here

*18/ Who decides which patients are included in your post-ICU follow-up program? Please select one or more answer below

A member of the ICU team (please specify below)

Other hospital specialists

The general practitioner

Other (please specify below)

+ free text : please specify here

*19/ When do you include patients in your post-ICU follow-up program? Please select one or more answer below

At ICU admission

During the ICU stay

At ICU discharge

After ICU discharge, while still in hospital

After hospital discharge

Other (please specify below)

+ free text : please specify here

*20/ When is the first follow-up appointment scheduled for? Please select a single answer

At 1 month after ICU discharge

At 2 months after ICU discharge

At 3 months after ICU discharge

At 6 months after ICU discharge

At 12 months after ICU discharge

Other (please specify below)

+ free text : please specify here

*21/ What is the goal of your follow-up? Please select one or more answer below

PICS screening

To ensure there are no other outstanding medical issues

Guidance on PICS management

Therapeutic patient education

Pharmacological treatment management

Discussion about treatment (de)escalation plan in case of further ICU readmission

PICS-F screening

Guidance on PICS-F management

Formal feedback to ICU teams

Clinical research

Other (please specify below)

+ free text : please specify here

*22/ Is each patient scheduled for one or more visit? Please select a single answer

A single visit

>> If this answer selected: go to question 24

More than one visit is systematically planned (please specify below how many	visits)

More than one visit is planned only if necessary

>> If one of the last 2 answers selected: go to question 23

+ free text : please specify here

*23/ When is the last follow-up visit usually scheduled? Please select a single answer

At 3 months after ICU discharge

At 6 months after ICU discharge

At 9 months after ICU discharge

At 12 months after ICU discharge

At 18 months after ICU discharge

At 24 months after ICU discharge

Other (please specify below)

+ free text : please specify here

*24/ How can patients participate to the post-ICU follow-up? Please select one or more answer below

In-person visit

Visit by telephone

Visit by video-link

Questionnaires sent as text (please specify below)

Dedicated follow-up medical app (please specify below)

Other (please specify below)

+ free text : please specify here

*25/ In your experience, what is the most frequent disability you usually detect in ICU survivors?

*26/ According to the patients you follow up, what do they feel is the most important disability that negatively impacts their recovery?

*27/ During the follow-up, do you explore disabilities other than physical dysfunction, cognitive dysfunction, anxiety, depression and post-traumatic stress disorders?

YES (please specify below) / NO

+ free text : please specify here

*28/ Are ICU diaries routinely implemented in your ICU?

YES / NO

>> if NO: go to page 7 and question 31

>> if YES: go to question 29 and 30

*29/ To whom are ICU diaries usually offered? Please select a single answer below	All patients admitted in ICU without any selection criteria

Patients with an anticipated prolonged ICU stay

Patients admitted to ICU for a long period of time (please specify below)

Patients with altered consciousness

Other (please specify below)

+ free text : please specify here

*30/ How and when is the ICU diary returned to the patient? Please select one or more answer below

In hospital alone without a specific support from the ICU team

In hospital alone, with specific support offered by the ICU team if required

At the post-ICU follow-up consultation

With the general practitioner

Other (please specify below)

+ free text : please specify here

Page 7

*31/ Which healthcare providers are involved in the post-ICU follow-up? Please select one or more answer below

physicians

nurses

advanced nurse practitioners

physiotherapists

psychologists

dieticians

pharmacists

other (please specify below)

+ free text : please specify here

*32/ Is a member of your ICU team involved in the post-ICU follow-up?

YES / NO

*33/ Do the healthcare professionals involved in the post-ICU follow-up receive dedicated training about post-ICU outcomes?

YES / NO

>> If YES: go to question 35

>> If NO: go to question 36

34/ Please specify the type of training they receive

35/ Please detail the type of training you think they should receive

*36/ In your opinion, are specialists and healthcare providers who treat or rehabilitate ICU survivors in hospital adequately trained in post-ICU issues?

YES / NO

+ free text

Page 8

*38/ Does the format of your follow-up align with specific guidelines?

YES (please specify below) / NO

+ free text : please specify here

>> if YES: go to question 40

>> If NO: go to question 39

*39/ Would you change the format of your follow-up to align with dedicated guidelines if some were published?

YES / NO

*40/ In your hospital is there any follow up provided in any transition phase?

No

Yes, in the transition phase between ICU and hospital general ward (please specify below)

Yes, in the transition phase between hospital and a rehabilitation centre (please specify below)

Yes, in the transition phase between hospital and home (please specify below)

+ free text: please specify who does this in-hospital follow-up and how it is performed

*41/ How do you collaborate with the primary care services in term of post-ICU follow-up? Please select one or more answer below

None

The general practitioner receives a copy of the ICU discharge summary

The general practitioner receives the report following the follow-up visit

The outcome of the follow-up visit is systematically discussed with the general practitioner

The primary care services oversee post-ICU care implementation

Other (please specify below)

+ free text : please specify here

*42/ In your opinion, do general practitioners and primary care providers have enough understanding of post-ICU health issues?

YES / NO

>> if NO: go to question 42

>> if YES: go to question 43

*43/ What should we do to increase general practitioners and primary care providers knowledge about post-ICU issues?

*44/ Do you collect the data related to your post-ICU follow-up? Please select a single answer

No

Yes, in a local database

Yes, in a regional registry

Yes, in a national registry

Yes, other (please specify below)

+ free text : please specify here

>> If YES, go to question 45 then 46

>> if NO: go to question 46

45/ Please detail the collected data

*46/ Do you plan to continue your follow-up program for the next few years?

YES / NO (if NO, please specify why below)

+ free text : please specify here

*47/ Who funds your post-ICU follow-up? Please select one or more answer below

Your hospital

The Government

Health insurance companies

Patients

Private donators

Other (please specify below)

+ free text : please specify here

Page 9

*48/ In your opinion, what are the perceived benefits of the post-ICU follow-up in your hospital? Please grade the answer as minor / moderate/ important

Patient satisfaction	minor / moderate/ important

Improvement in patient health status	minor / moderate/ important

Relative satisfaction	minor / moderate/ important

Hospital readmission prevention	minor / moderate/ important

ICU team satisfaction	minor / moderate/ important

ICU quality of care improvement	minor / moderate/ important

Positive impact on hospital image	minor / moderate/ important

Financial benefits for the hospital	minor / moderate/ important

General practitioner satisfaction	minor / moderate/ important

Other (please specify below)

+ free text : please specify here

*49/ Has the therapy/daily practice approach changed in your ICU due to the follow-up data?

YES / NO

+ free text : please specify here

*50/ From a general point of view, what progress needs to be made to improve the care of ICU survivors in your country? Please select one or more answer below

Nothing

Allowing each patient to have access to a post-ICU screening by experts

Allowing each patient to have access to a post-ICU rehabilitation

Reducing PICS screening and rehabilitation costs for patients

Setting up an ICU survivors association

Other (please specify below)

+ free text : please specify here

51/ In your opinion, how can ESICM help improve the care of ICU survivors in Europe?

Page 10

Thank you very much for your collaboration!

We would appreciate staying in contact with you, in case there is anything that requires clarification.

If you agree, please write your email address here: …

2/ ICU-S questionnaire

Page 1

CORFU survey

Access to and perceived benefits of post-ICU follow-up in Europe: the perspective of ICU survivors

**Dear Patients,**

**We kindly invite you to participate in a survey titled “CORFU survey”.**

**This survey, endorsed by the European Society of Intensive Care Medicine (ESICM), is run by its HSRO section, that is dedicated to outcome research.**

**This survey aims to depict the current practices in terms of post-ICU follow-up**

**and post-intensive care syndrome screening across Europe and to assess the perceived benefits of PICS-targeted post-ICU follow-up.**

**The answers to these questionnaires will be helpful to prepare further dedicated recommendations or expert consensus on the topic.**

**The survey takes about 2 to 10 minutes to complete.**

We are seeking participation from ICU survivors living in Europe. ***If you are not a European ICU survivor, please do not complete the survey.***

**Participating in this survey does not expose you to any risk: participation is voluntary and anonymous.**

This research has been reviewed by an Ethical Committee of the University Hospital of Liège (Belgium). Refusal or choice not to participate will not result in any penalty.

The principal investigator, the research team and the sponsor undertake to comply with the regulations on the protection of natural persons in relation to the processing of personal data. The information collected in this survey will be stored securely as long as there is a need for this research purpose. The collected data will not be used or distributed for other future projects.

If you have any questions about your rights as a research participant at any time before, during or after participation, please contact the study team (principal investigator, Pr Anne-Françoise ROUSSEAU, afrousseau@chuliege.be).

**We thank you for your collaboration,**

**On behalf of the HSRO section,**

**Anne-Françoise Rousseau, Michael Beil and Susannah Leaver**

Would you like to proceed with this anonymous survey ?

YES / NO

If NO: go to page 6

Page 2

Please read the following abbreviations and definitions before proceeding with the survey:

ICU: intensive care unit

specialised wards in the hospital, where a multidisciplinary team, made up of highly skilled intensive care doctors, nurses, and other healthcare providers, ensure 24-hour critical care for patients at risk of or presenting with one or more organ failure.

PICS: post-intensive care syndrome

new onset or worsening of impairment(s) in physical, cognitive, and/or mental health that arise after the ICU and persists beyond hospital discharge, affecting daily function and quality of life. PICS can include post-traumatic stress disorder, but is not limited to this.

Post-ICU follow-up: consultations with healthcare providers especially aimed to monitor the evolution after an ICU stay

Please remember that the questionnaire is about the follow-up after the ICU stay and not the ICU stay itself or the stay in another unit in a hospital.

Don't worry about the question numbers; your responses will determine the order.

* mandatory questions

Page 3

* 1/ Your initials (the first two letters of your mother’s first name and the first two letters of your father’s surname)

*2/ Where are you from (country)?

*3/ How old are you?

*4/ Have you been a patient on ICU during the 2 last years?

YES / NO

>> if YES: go to page 4 and question 5

>> if NO,: go to page 6

Page 4

*5/ In what type of hospital was your ICU admission? Please select a single answer

University / teaching hospital

Non university / teaching hospital

I don’t know

*6/ Where was this hospital (city / town)?

*7/ What was the name of this hospital?

*8/ How long did you stay in ICU? (days)

*9/ Did you receive any information about the post-intensive care syndrome (PICS) during or after your ICU stay?

YES / NO / I don’t remember

>> if YES: go to questions 10 and 11 and then 15

>> If NO / I don’t remember: go to question 12

10/ Please specify the format, and the content of the received information

*11/ Do you think this information was useful?

YES / NO

+ free text: please specify

*12/ Do you think this information would have been useful?

YES / NO

>> If YES: go to questions 13 and 14 and then 15

>> if NO: go to question 15

13/ In your opinion, what would have been the best format to receive this information in?

14/ When would have been the most useful time to receive this information?

*15/ Did you benefit from a dedicated post-ICU follow-up, aiming to detect complications specific to being an ICU survivor?

YES / NO

>> if NO: go to question 16

>> if YES: go to page 5 and question 17

*16/ Would such follow-up have been useful for you?

YES (please explain why below) / NO (please explain why below)

+ free text: please explain

END of the survey: go to page 6

Page 5

*17/ Who organized this follow-up? Please select one or more answer below

The ICU team

The general practitioner / family physician

Another healthcare provider in the hospital

Another healthcare provider outside the hospital

Other (please specify below)

I don’t know

+ free text: please specify

*18/ When was the first follow-up appointment? Please select a single answer

In hospital after ICU discharge

At 1 month after ICU discharge

At 2 months after ICU discharge

At 3 months after ICU discharge

At 6 months after ICU discharge

At 12 months after ICU discharge

Other (please specify below)

+ free text: please specify

*19/ Which of the following were explored? Please select one or more answer below

My muscle strength

How I eat

My mood

My mental abilities (attention, remembering, problem solving, …)

My swallowing abilities

How I sleep

How I am dependent for the daily living activities

My quality of life

Blood tests

Other (please specify below)

I don’t remember

I don’t understand the question

+ free text: please specify

*20/ Were you given an ICU diary after ICU discharge? (An ICU diary is a paper or electronic diary that is written by the relatives and the nurses, to keep a narrative of patient’s ICU stay)

YES / NO / I don’t know what an ICU diary is

>> if YES: go to question 21, 22, 23 and then 24

>> if NO / I don’t know: go to question 24

*21/ Did you already read your ICU diary?

YES / NO

>> if YES, go to questions 22 and 23 and then 24

>> if NO: go to question 24

*22/ How did you discover its content? Please select a single answer below

I read it alone

I read it with my loved ones

I read it with a member of the ICU team

I read it with a psychologist

I read it with my general practitioner

Other (please specify below)

+ free text: please specify

*23/ Did you find it beneficial? Please select one answer below

It had a negative impact on me

It had no impact on me

It had a positive impact on me

*24/ During the follow-up visit, did you discuss your time in ICU, confusion, discomforts or suffering you may have experienced during the ICU stay? Please select a single answer

Yes

No, I didn’t want to

No, I didn’t have the opportunity

No, for another reason (please specify)

+ free text: please specify

>> if NO: go to question 25 and then 27

>> if YES: go to question 26 and then 27

*25/ Would you have liked to have expressed yourself / discuss about your experience in ICU?

YES / NO

*26/ Were all your issues addressed during the follow-up?

YES / NO

>> if YES: go to question 28

>> if NO: go to question 27 and then 28

27/ Please specify the issues you would have liked to be addressed

*28/ In your opinion, what was the effect of the post-ICU follow-up on you? please select a single answer

I got significantly worse

I got slightly worse

I perceived no effect

I got slightly better

I got significantly better

+ please explain why this follow-up had such an impact on you

*29/ In your opinion, what should be the objective of a well conducted specific follow-up after an ICU stay? Please select one or more answers below

Ensure patients do not feel abandoned in their post-ICU recovery

Ensure relatives do not feel abandoned in their post-ICU recovery

Provide access to dedicated care for specific post-ICU symptoms

Improve patient’s health status

Improve patient’s quality of life

Provide information about what happened during the ICU stay to the patient

Provide information about what happened during the ICU stay to the relatives

Discuss about advanced directives for future medical decisions

Use patient’s feedback to improve critical care for future patients

Other (please specify below)

+ free text: please specify

30/ In your opinion, how could the medical community improve the care of ICU survivors?

Page 6

Thank you very much for your collaboration!

We would appreciate staying in contact with you, in case there is anything that requires clarification.

If you agree, please write your email address here: ….

*(not mandatory)*
