# Supplementary material for: Access to and perceived value of post-ICU follow-up: an international cross-sectional survey of ICU professionals and adult critical illness survivors
Source: Ann Intensive Care. 2026 Aug 26;16:100141. doi: 10.1016/j.aicoj.2026.100141 (PMC13564131; doi:10.1016/j.aicoj.2026.100141)
Supplement: Supplementary file 3 [file mmc3.docx]

**Access to and perceived value of post-ICU follow-up:**

**an international cross-sectional survey of ICU professionals and**

**adult critical illness survivors.**

Supplemental Material

Supplemental Table 1: Geographical distribution of the ICU-HP survey participants across countries. The table details the number of respondents, the number of different represented ICUs, and the respective number of offered post-ICU follow-up clinic in each category.

| Countries | Survey respondents | Respondents with an available post-ICU follow-up clinic | Different represented ICUs | ICUs with an available post-ICU follow-up clinic |
| --- | --- | --- | --- | --- |
| Albania | 1 | 0 | 1 | 0 |
| Argentina | 4 | 1 | 4 | 1 |
| Australia | 2 | 2 | 1 | 1 |
| Austria | 18 | 5 | 16 | 5 |
| Bangladesh | 1 | 0 | 1 | 0 |
| Belgium | 6 | 1 | 6 | 1 |
| Bolivia | 2 | 0 | 2 | 0 |
| Bosnia-Herzegovina | 1 | 0 | 1 | 0 |
| Brazil | 2 | 0 | 2 | 0 |
| Chile | 12 | 1 | 10 | 1 |
| Croatia | 4 | 2 | 3 | 2 |
| Denmark | 7 | 7 | 4 | 4 |
| Estonia | 2 | 0 | 2 | 0 |
| France | 49 | 27 | 45 | 22 |
| Germany | 8 | 1 | 7 | 1 |
| Greece | 7 | 1 | 5 | 1 |
| Hungary | 1 | 0 | 1 | 0 |
| India | 23 | 11 | 22 | 11 |
| Indonesia | 1 | 0 | 1 | 0 |
| Ireland | 6 | 6 | 3 | 3 |
| Israel | 4 | 1 | 2 | 1 |
| Italy | 4 | 2 | 4 | 2 |
| Japan | 1 | 0 | 1 | 0 |
| Lithuania | 1 | 0 | 1 | 0 |
| Luxemburg | 1 | 0 | 1 | 0 |
| Malta | 1 | 1 | 1 | 1 |
| Mexico | 3 | 1 | 2 | 1 |
| Moldova | 7 | 2 | 4 | 1 |
| The Netherlands | 6 | 6 | 6 | 6 |
| New Zeeland | 1 | 1 | 1 | 1 |
| Norway | 4 | 1 | 2 | 1 |
| Poland | 15 | 4 | 14 | 4 |
| Portugal | 8 | 8 | 6 | 6 |
| Romania | 17 | 2 | 17 | 2 |
| Saudi Arabia | 1 | 1 | 1 | 1 |
| Spain | 8 | 5 | 8 | 5 |
| Sweeden | 2 | 2 | 2 | 2 |
| Switzerland | 9 | 5 | 7 | 4 |
| Tanzania | 1 | 0 | 1 | 0 |
| Turkey | 8 | 4 | 8 | 4 |
| United Kingdom | 14 | 14 | 13 | 13 |
| United State of America | 2 | 1 | 2 | 1 |
| Uruguay | 1 | 0 | 1 | 0 |

Supplemental Table 2: Patients’ suggestions on how the medical community should improve the care of ICU survivors (open question)

Suggestions

Provide access to psychological support

Ensure that healthcare professionals actively listen to patients

Provide a clear and structured post-ICU care pathway

Identify a care coordinator in order to know whom to contact throughout the post-ICU pathway

Make a post-ICU follow-up accessible to all ICU survivors (without restrictive eligibility criteria)

Provide early and regular follow-up appointment to avoid feelings of abandonment after ICU discharge

Provide follow-up as early as transition between ICU and hospital ward

Actively involve relatives

Supplemental Figure 1: Expected usefulness of dedicated post-ICU follow-up among ICU-S who did not benefit from follow-up.

*Numbers displayed within / besides the bars indicate the proportion of ICU-HP (out of the total sample of 78 respondents) who reported each item.*

*ICU: intensive care unit*

Supplemental Figure 2: Characteristics of post-ICU follow-up programs according to ICU-HP involved in or aware of the program in their institution.

*- patient selection criteria (close-ended question with multiple responses allowed)*

*- timing of program initiation (close-ended question with multiple responses allowed)*

*- timing of the first follow-up visit (close-ended question with single response allowed)*

*- visit modalities (close-ended question with multiple responses allowed)*

*- number of visits (close-ended question with single response allowed)*

*- timing of the final follow-up visit (close-ended question with single response allowed)*

*Numbers displayed within / besides the bars indicate the proportion of ICU-HP (out of the total sample of 113 respondents) who selected each answer.*

*ICU: intensive care unit*

Supplemental Figure 3: Characteristics of post-ICU follow-up programs according to ICU-S

*- timing of the first follow-up appointment (close-ended question with single response allowed)*

*- domains explored during follow-up (close-ended question with multiple responses allowed)*

*Numbers displayed besides the bars indicate the proportion of ICU-HP (out of the total sample of 62 respondents) who selected each answer.*

*ICU: intensive care unit*

Supplemental Figure 4: The most frequent detected disability during post-ICU follow-up, according to 78 ICU-HP respondents (open-ended question)

*Numbers displayed within / besides the bars indicate the proportion of ICU-HP (out of the total sample of 78 respondents) who selected each answer.*

*HP: healthcare provider; ICU: intensive care unit*

Supplemental Figure 5: Impact of post-ICU follow-up on ICU survivors and their rationale (open-ended question).

*Data are expressed as counts and percentages.*

*ICU: intensive care unit*

Supplemental Figure 6: Collaboration with primary care services in term of post-ICU follow-up (close-ended question with multiples responses allowed).

*Numbers displayed besides the bars indicate the proportion of ICU-HP (out of the total sample of 80 respondents) who selected each answer.*

*GP: general practitioner; ICU: intensive care unit*

Supplemental Figure 7: Sources of funding for post-ICU follow-up programs (close-ended question with multiples responses allowed).

*Numbers displayed besides the bars indicate the proportion of ICU-HP (out of the total sample of 80 respondents) who selected each answer.*
